# Maximizing the positive and minimizing the negative: Social media data to study youth mental health with informed consent

**DOI:** 10.3389/fpsyt.2022.1096253

**Published:** 2023-01-10

**Authors:** Daniel Leightley, Amanda Bye, Ben Carter, Kylee Trevillion, Stella Branthonne-Foster, Maria Liakata, Anthony Wood, Dennis Ougrin, Amy Orben, Tamsin Ford, Rina Dutta

**Affiliations:** ^1^Department of Psychological Medicine, Institute of Psychiatry, Psychology and Neuroscience, King's College London, London, United Kingdom; ^2^Department of Biostatistics and Health Informatics, Institute of Psychiatry, Psychology and Neuroscience, King's College London, London, United Kingdom; ^3^Health Service and Population Research Department, Institute of Psychiatry, Psychology and Neuroscience, King's College London, London, United Kingdom; ^4^Lived Experience Advisor, London, United Kingdom; ^5^School of Electronic Engineering and Computer Science, Queens Mary University of London, London, United Kingdom; ^6^Qntfy, Arlington, VA, United States; ^7^Centre for Psychiatry and Mental Health, Wolfson Institute of Population Health, Queen Mary University of London, London, United Kingdom; ^8^Department of Child and Adolescent Psychiatry, Institute of Psychiatry, Psychology and Neuroscience, King's College London, London, United Kingdom; ^9^MRC Cognition and Brain Sciences Unit, Cambridge, United Kingdom; ^10^Department of Psychiatry, University of Cambridge, Cambridge, United Kingdom; ^11^South London and Maudsley NHS Foundation Trust, London, United Kingdom

**Keywords:** social media, data protection, research ethics, risk, Facebook

## Abstract

Social media usage impacts upon the mental health and wellbeing of young people, yet there is not enough evidence to determine who is affected, how and to what extent. While it has widened and strengthened communication networks for many, the dangers posed to at-risk youth are serious. Social media data offers unique insights into the minute details of a user's online life. Timely consented access to data could offer many opportunities to transform understanding of its effects on mental wellbeing in different contexts. However, limited data access by researchers is preventing such advances from being made. Our multidisciplinary authorship includes a lived experience adviser, academic and practicing psychiatrists, and academic psychology, as well as computational, statistical, and qualitative researchers. In this Perspective article, we propose a framework to support secure and confidential access to social media platform data for research to make progress toward better public mental health.

## Introduction

The viewing of self-harm-related images and posts have been cited as factors in the suicide of young people across the world (1). Not all social media use is detrimental to mental health however, and it is increasingly harnessed to provide support and even suicide prevention strategies (2, 3).

Testimony to US Congress in October 2021 by a former Facebook employee, provided evidence that the social media platform concealed internal research findings regarding the potential negative impact of its Instagram platform on some youth (4). Furthermore, social media platforms have been blocking access to data by external researchers, potentially delaying the development of life-saving advances and discoveries (5). Social media data, for example, offers the scientific community unique insights into the details of a person's digitally mediated life. Near-real-time access to data paired with the informed consent of the individual, could provide many positive opportunities in a clinical setting.

Following the Cambridge Analytica scandal of 2018, where the personal data of millions of Facebook users was harvested without their consent, the platform tightened access to its Application Programming Interface (API), which served as the main tool by which legitimate researchers collected behavioral and digital trace data (6). Facebook's current complex and lengthy guidelines for data access are aimed at commercial organizations and centred on a notion of “e*nhancing user experience*,” including by means of targeted personal advertising (7). Research does not usually intend to improve the individual “user experience,” but instead has wider societal implications. It is important to note that in light of prior misuses of Facebook's API, all use of the API is required to be verified by Facebook and must meet Facebook's own guidelines.

This difference in focus by researchers can result in lack of access to platform products offered widely to the commercial sector, such as the open authentication protocol allowing login and access to user content *via* a user's Facebook login. This disparity in data accessibility between commercial and academic interests, with commercial gain prioritized over research for public benefit, raises a vital question for scientific research and data ownership. How might we conduct independent, academic research into the impact of social media use on behavioral health and wellbeing when access to data is so limited?

Researchers who try to develop expensive and difficult to maintain bespoke data collection pipelines (i.e., systems designed to regularly extract and store data from consented users), compliant with the terms of service specified by a platform to collect publicly available data, are often unsuccessful. Their requests are reviewed by the platform, and a decision is made stating that the terms are violated as they do not enhance the “*user experience.”* There is no independent review or appeals process external to the organization with limited engagement and consultation with academic or lived experience researchers to develop systems that meet the needs of all parties.

Moreover, researchers encounter increasingly negative scenarios when they attempt to access social media data from fully consented and ethically approved studies with active participants. Even when informed user consent is carefully documented, social media platforms do not permit (or have technical roadblocks) to data access. As an example, our US colleagues created a system for the consented donation of online data (OurDataHelps.org), to screen for suicide risk and varied mental health diagnoses using natural language processing (3, 8). By January 2021, more than 4,000 individuals had provided self-report mental health data and social media content, yet the agreed access and collection of data was revoked by the platforms. There were neither complaints by the participants nor breaches of data, the platforms simply made it impossible for this project to continue despite a long track record of qualified support of peer-reviewed research by leading Universities.

For us the answer is simple. While users must provide consent to social media platforms for the processing of their personal data, it should be for the user to decide how their other data is processed and used (9). Barriers should not be placed in the way of users making informed decisions about this.

Such a view is supported by legislation and data regulation. In the European Union and the European Economic Area, the use of personal data is regulated by the General Data Protection Regulation 2016/679 - commonly known as GDPR - which poses a number of conditions under which data processing may be considered lawful. The most transparent way for academics to process data and comply with the regulations is by obtaining informed consent.

GDPR also specifies that data processing may be lawful if it is “necessary for the performance of a task carried out in the public interest.” Since it is generally straightforward to defend academic research by accredited Universities as pertaining to public interest, data collection, analysis, and publication for scientific purposes is protected by the GDPR. This is particularly the case when participants have provided informed consent for the use of their data. Yet social media platforms are using presumed incompatibility with data privacy and accessibility as a justification to deflect or deny qualified researcher access requests.

The default position of academic study is to rely heavily on self-reported social media use which is known to be an inaccurate proxy for logged media use (10). Alternatively, participants are burdened with the unwieldy task of requesting and accessing a copy of their data and providing this to researchers (11). The systems the platforms provide to the user, while compliant with the law, are not user friendly for this purpose, which presents the researcher with complications for data completeness and participant retention. It is imperative that we move beyond self-report and utilize behavioral data from platforms including Apple iOS, Google Android, Facebook, Instagram, TikTok, Twitter and YouTuben to understand more objectively how young people are interacting online and how and when this affects their mental wellbeing, in ways that are acceptable to participants.

To address the concerns of the research community about users' safety and security, the UK government's Online Harms White Paper (12), released in 2020, pledged to provide “researchers with access to company data to support research into online harms.” The government proposal also included a 2% “turnover tax” levy on the UK revenues of major technology companies to fund independent research and training packages for clinicians, teachers and other professionals working with children and young people, though this has since been reversed with the Organization for Economic Co-operation and Development (OECD) taxation agreement earlier this year.

Neither recommendation has been realized. Instead, both proposals have been removed from the delayed Online Safety Bill, to be replaced only by a requirement for the government-approved regulatory Office of Communications (OFCOM) to prepare a report explaining how independent researchers are “(a)…currently able to obtain information from providers of regulated services to inform their research, (b) exploring the legal and other issues which currently constrain the sharing of information for such purposes, and (c) assessing the extent to which greater access to information for such purposes might be achieved” (13).

## A framework of recommendations

The UK government's Online Harms White Paper's (12) suggested introduction of a voluntary best practice frameworks has not been included in the Draft Online Safety Bill (13), and would not ensure social media platforms met their ethical responsibilities (e.g., data protection, participant health and safety).

Over the last 20 years, social media platforms have been able to develop their own rules as to what, how and why an individual, organization or researcher can access user data. Often these rules change without notice, without prior notification and irrespective of the potential harm this may cause. Therefore, we are proposing a framework to facilitate regulated and monitored access for researchers to social media platform data in order to make long-term progress toward public mental health.

**Figure 1:**
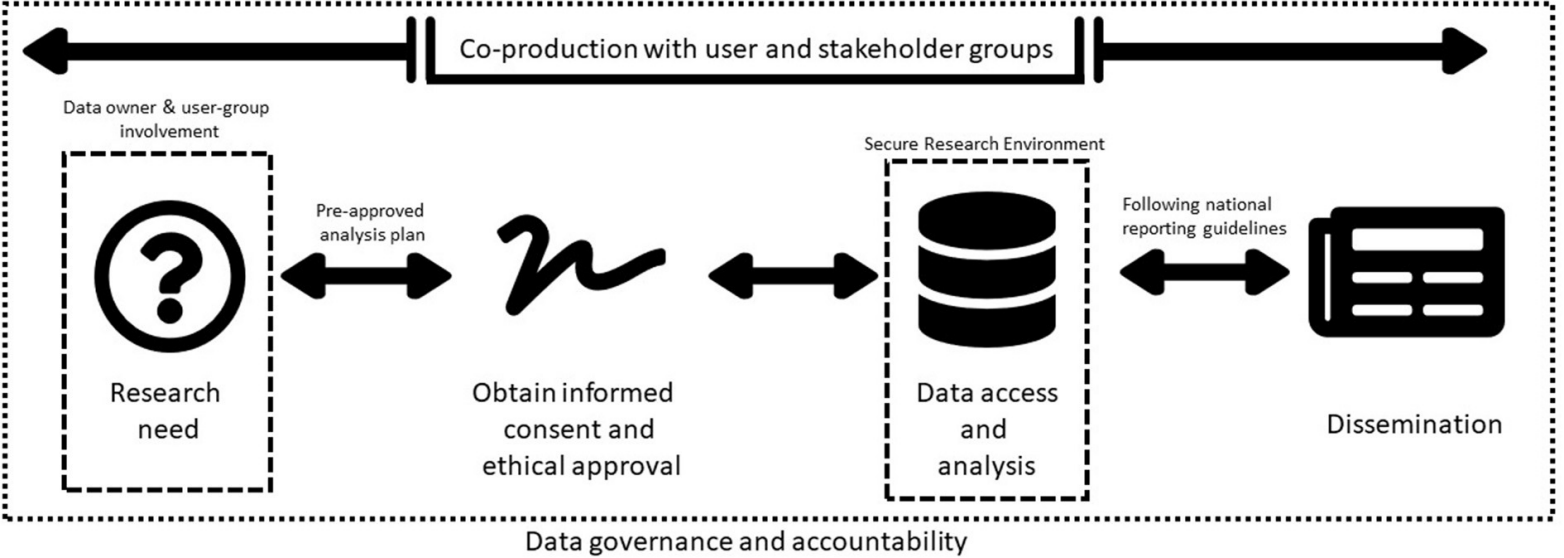
Proposed framework to regulate and monitor researchers' access to social media platform data.

Our framework has four core elements and a cross-cutting theme integral to each stage (refer to Figure 1).

Co-production with user and stakeholder groups is the cross-cutting theme embedded and incorporated into each element of the framework. Using established public and patient involvement standards (e.g., UK Standards for Public Involvement, NIHR: https://sites.google.com/nihr.ac.uk/pi-standards/home), researchers should work collaboratively with those with lived experience, carers and members of the public to first identify the research priorities and then co-produce research protocols and methods.

### Research need

Qualified researchers at accredited Universities intending to use social media data to understand and improve youth mental health, should co-produce their research with patients, carers and members of the public. Researchers shall undertake user-centred engagement in line with established public and patient involvement model criteria throughout the study; justify the rationale for data access and engage data owners in the proposed research (14). The data owner and user-group should also review and approve analysis plans to ensure the approach is acceptable, ethically-sound, feasible and of value.

### Ethical approval and informed consent

Participants should always be empowered to understand why and how their data will be used for research. This should be in accessible and acceptable formats which user groups co-produce with researchers. Ethically-approved informed consent procedures will state exactly what is being collected, how it will be processed and how results will be reported. This will include clear accessible guidance on how data will be managed following GDPR.

### Data access and analysis

Certain social media data inherently cannot be fully anonymised due to free-text and use of images/videos. Therefore, robust data governance guidelines and well-defined individual institutional accountability should be established, on a par with current protocols for medical research. This would include analyzing data in a Secure Research Environment (SRE), where access is intensively monitored and controlled. Data owners should agree data sharing agreements with SRE providers. Exemplars in the UK include the Office for National Statistics Secure Research Service (SRS), which records each interaction with the data and restricts what researchers might do with the data. Having a trained service user group with lived experience involved in qualitative data analysis can realign researchers' misinterpretations and challenge the ways in which findings are reported adding value to the products of research analysis.

### Open dissemination

A Registered Report format is recommended, which *via* standardization, would improve the peer review process to be conducted before data collection and public dissemination of research findings. Lived experience advisers or service user researchers should be included in the authorship of documents, briefings and research papers arising. This would promote better accessibility, transparency and collaboration for the public, academic community and other interested groups in accordance with the Open Science Framework.

## Conclusions

Gaining informed consent for social media data access to study youth mental health has the potential for significant benefits in public mental health. Data collected *via* social media platforms provide us with a unique opportunity to gather vital insights into participants' actions and activities. This unparalleled access will help researchers understand the intricate social constructs of user interactions, perceptions, mental state and health.

At present, the poorly defined term “enhancing user experience” is the main factor that social media platforms apply in determining if access is granted. However, accredited researchers' use of social media platform data does not usually improve user “experience” in the commercial sense, rather it has the potential for wider positive public benefits which are unlikely to be of primary interest for social media platforms.

Tackling harmful and negative content is a global problem, but one solution is to provide access for researchers to understand the problem. It is important that we unlock social media data's potential for research and leverage the data for societal good. We hope this framework will be a “call to action” to stimulate social media platforms, policy makers and researchers to make positive changes by collaborative working.

## Data availability statement

Publicly available datasets were analyzed in this study. This data can be found at: Perspective article. Original contribution relates to https://gtr.ukri.org/projects?ref=MR%2FS020365%2F1.

## Author contributions

DL, AB, BC, KT, SB-F, ML, AW, TF, and RD contributed to conception and planning of this perspective article. DL and RD wrote the first draft of the manuscript. All authors contributed to sections of the manuscript according to expertise, editing, revision, and approved the submitted version.
